# Early differentiation of viral encephalitis and autoimmune encephalitis by clinical and basic laboratory data

**DOI:** 10.3389/fneur.2026.1827372

**Published:** 2026-10-13

**Authors:** Tim Steinberg, Andreas Hierl, David Freudenstein, De-Hyung Lee, Ralf A. Linker, Markus Bauswein, Hendrik Hartmann, Barbara Schmidt, Klemens Angstwurm

**Affiliations:** 1 Department of Neurology, University Hospital Regensburg, Regensburg, Germany; 2 Department of Neurology and Neurophysiology, University Hospital Augsburg, Augsburg, Germany; 3 Department of Psychiatry, Bezirksklinikum Wöllershof, Störnstein, Germany; 4 Institute of Clinical Microbiology and Hygiene, University Hospital Regensburg, Regensburg, Germany; 5 Center for Clinical Studies, University Hospital Regensburg, Regensburg, Germany

**Keywords:** autoimmune encephalitis, cerebrospinal fluid, herpes simplex virus encephalitis, tick-borne encephalitis, varicella zoster encephalitis, viral encephalitis

## Abstract

**Background and Objectives:**

Timely differentiation between autoimmune and viral causes of encephalitis is critical for guiding disease-specific treatment. This study aimed to develop a scoring system to facilitate early (< 6 h after presentation) differentiation between viral encephalitis (VE) and autoimmune encephalitis (AE).

**Methods:**

We performed a retrospective, single-center analysis of all patients diagnosed with encephalitis for any etiology at the University Hospital Regensburg between January 1st, 2004 and December 31st, 2020. Based on patient history, clinical findings, and cerebrospinal fluid (CSF) parameters, we developed a scoring system to differentiate between VE and AE.

**Results:**

A total of 434 cases with encephalitis were included, of which 171 had *Herpes simplex* virus encephalitis, V*aricella zoster* virus encephalitis, tick-borne encephalitis or autoimmune encephalitis. Based on a homogeneous subgroup of 108 cases of VE and 44 cases of AE, we used a logistic regression model to derive a score including fever, headache, CSF white blood cell count, CSF lactate concentration, and CSF protein concentration for early differentiation. The score showed very good discrimination (AUC 0.94, 95% confidence interval (CI): 0.90–0.99), with a specificity of 87.0% and sensitivity of 88.6% in our cohort.

**Discussion and conclusion:**

Our scoring system might help to identify patients with VE or AE at presentation. Further validation is needed.

## 1 Introduction

The incidence of viral encephalitis (VE) excluding epidemics is between 1.5 and 7 cases/100,000 inhabitants/year (1). In Bavaria, tick-borne encephalitis (TBE) is endemic and causes a relevant proportion of those cases (incidence 0.7–2.0/100,000 inhabitants/year) (2). According to current literature, the etiology in patients with encephalitis is most likely infectious (42%), unknown (37%) and autoimmune (21%) (3). The primary diagnostic step in patients with suspected encephalitis is cerebrospinal fluid (CSF) analysis, especially regarding white blood cell (WBC) count as well as lactate, glucose and protein concentrations (4). Until microbiological results are obtained, antiviral therapy with acyclovir and antibacterial therapy is initiated (5). After confirmation of negative microbiological findings, autoimmune encephalitis (AE) is considered, and immunosuppressive therapy is started–resulting in a delay before specific treatment is administered. Delayed treatment, however, results in a worse prognosis in cases of AE (6). Additionally, false-negative microbiological results may occur at an early stage of VE, which may lead to premature termination of antiviral treatment (7). Therefore, there is a need for diagnostic tools that can be applied early to help distinguish between VE and AE and facilitate specific treatment. In this study, we compare clinical data and CSF findings in patients with the diagnosis of definite AE and definite VE in order to develop a scoring system to predict AE.

## 2 Methods

### 2.1 Patient selection

All cases treated at the neurological department of the University Hospital of Regensburg between January 1st, 2004 and December 31st, 2020 were screened for the diagnosis of encephalitis of any etiology. Encephalitis was diagnosed according to Venkatesan et al. (8). Patients were excluded if no CSF results were documented.

### 2.2 Patient analysis

AE was assumed, if a corresponding, specific antibody was found or the patient responded to immunosuppressive treatment (IST) (9). Diagnosis of *Herpes simplex* virus (HSV) or *Varicella zoster* virus (VZV) encephalitis was based on CSF polymerase chain reaction (PCR) or intrathecal synthesis of specific antibodies, and of tick-borne encephalitis based on specific immunoglobulin (Ig) M and IgG in serum, positive CSF/serum IgG antibody specificity index (ASI) or positive CSF PCR. Baseline parameters, patient history and clinical information (headache, fever >38 °C, altered neuropsychiatric status, focal neurological deficits, epileptic seizures), CSF results of the first lumbar puncture after symptom onset [including WBC count, lactate concentration, protein concentration, CSF/serum albumin quotient (as a measure of blood brain barrier disturbance), oligoclonal bands] were acquired. Altered neuropsychiatric status was defined as the presence of new cognitive deficits, personality changes, psychiatric symptoms, and/or impaired consciousness. For the development of our score, the cohort of VE consisted of the most frequent viral pathogens in our cohort (tick-borne encephalitis, HSV and VZV). For comparison, we included all cases of isolated AE of the central nervous system (CNS) with corresponding antineuronal or paraneoplastic antibodies.

### 2.3 Statistical analyses

Statistical analyses were conducted using SPSS Statistics, version 31 (IBM Corp., Armonk, NY, USA) and R, version 4.5.2 and the following R packages: *tidyverse, haven, car, logistf, rms, dcurves, pROC* (10–17). For descriptive statistics, absolute and relative frequencies were reported, along with measures of central tendency and dispersion (mean, standard deviation). Univariate logistic regression was used to assess differences in parameters in regard to the differentiation of AE and VE. Based on clinical relevance, early test availability, literature research and results of the univariate regression analysis, five parameters were chosen for the score. Cut-off values to dichotomize the metric parameters were defined using Receiver Operating Characteristic (ROC)–Curves. The associations between the predictors and AE were identified using Firth penalized logistic regression to reduce small-sample bias and to provide stable coefficient estimates in the presence of low events-per-variable ratios or complete separation. Internal validation was performed using bootstrap resampling (1,000 iterations). Multicollinearity was assessed with Variance inflation factor (VIF) analysis. Model performance was assessed by optimism-corrected discrimination [area under the receiver operating characteristic curve (AUC)], calibration (calibration slope and calibration plot), and the accuracy of predictions [Brier score (18)]. A point-based sum score was derived by scaling the regression coefficients. Coefficients were divided by the smallest coefficient and rounded to the nearest integer while preserving their relative weights. The diagnostic performance of the final score was evaluated by receiver operating characteristic analysis, with the optimal cut-off determined using the Youden index. Sensitivity, specificity, positive (PPV) and negative predictive values (NPV), positive (LR+) and negative likelihood ratio (LR–), and corresponding 95% confidence intervals (CI) were calculated. Clinical utility of the sum score was evaluated against the regression model by decision curve analysis [DCA (19)], comparing the net benefit of the sum score with treat-all and treat-none strategies across a range of threshold probabilities.

Because age at onset might provide predictive potential to discriminate AE and VE, but clinical results on its association are inconclusive (20), a sensitivity analysis with age at onset included in the regression model was performed.

## 3 Results

### 3.1 Cohort Description

A total of 434 patients were included for having encephalitis. Mean age was 54 ± 17 years (minimum 15 years, maximum 94 years), 33.9% were female. The distribution of different etiologies is presented in Figure 1. VE was identified in 152 patients (35%), autoimmune disease in 93 patients (21%), and other infectious causes in 29 patients (7%). Notably, the etiology remained unclear in 156 cases (36%). For more detailed analysis of the underlying pathogens and diseases, please refer to Supplementary Table 1.

**Figure 1:**
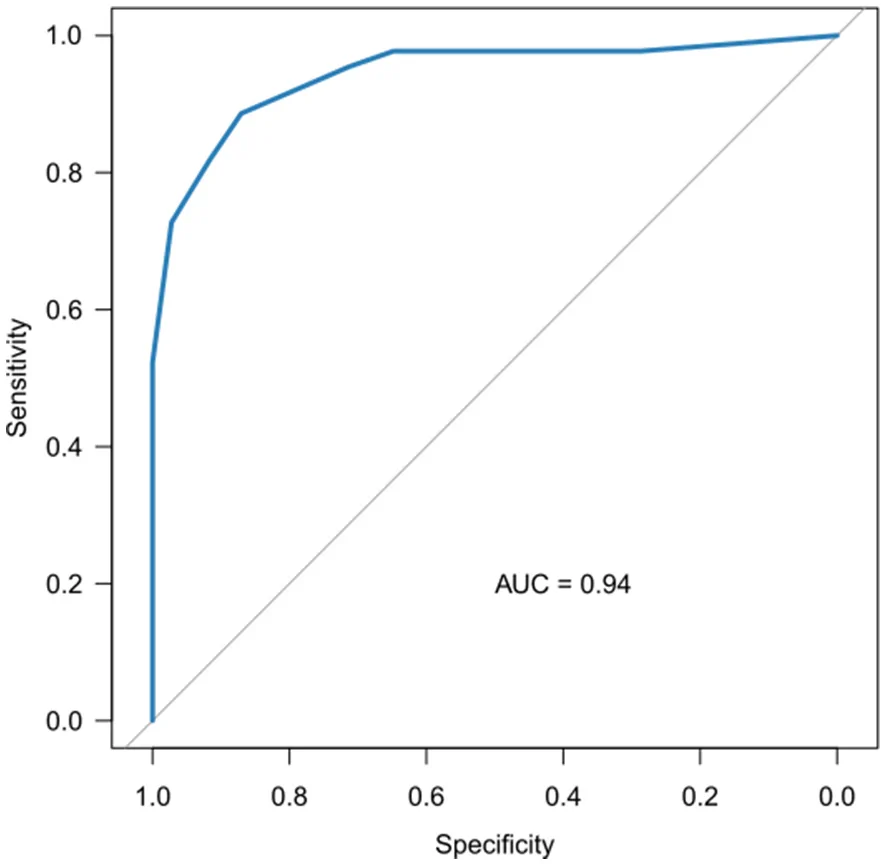
The distribution of encephalitis etiologies in our cohort is presented as absolute and relative frequencies, demonstrating that viral encephalitis and encephalitis of unknown origin were the most common entities, while a range of less frequent etiologies was also observed. HSV, *Herpes simplex* virus; VZV, *Varizella Zoster* virus.

To compare VE and AE, we aimed to include a cohort of well-defined cases and, therefore, selected the three largest subgroups of viral encephalitis: HSV encephalitis, VZV encephalitis, and TBE. For AE, we focused exclusively on isolated autoimmune diseases of the central nervous system, excluding cases associated with systemic autoimmune disorders or cases with suspected autoimmune encephalitis with no evidence of a specific antibody. 28 cases were excluded due to incomplete data, resulting in a total of 152 cases available for score development. For detailed information, see Figure 2.

**Figure 2:**
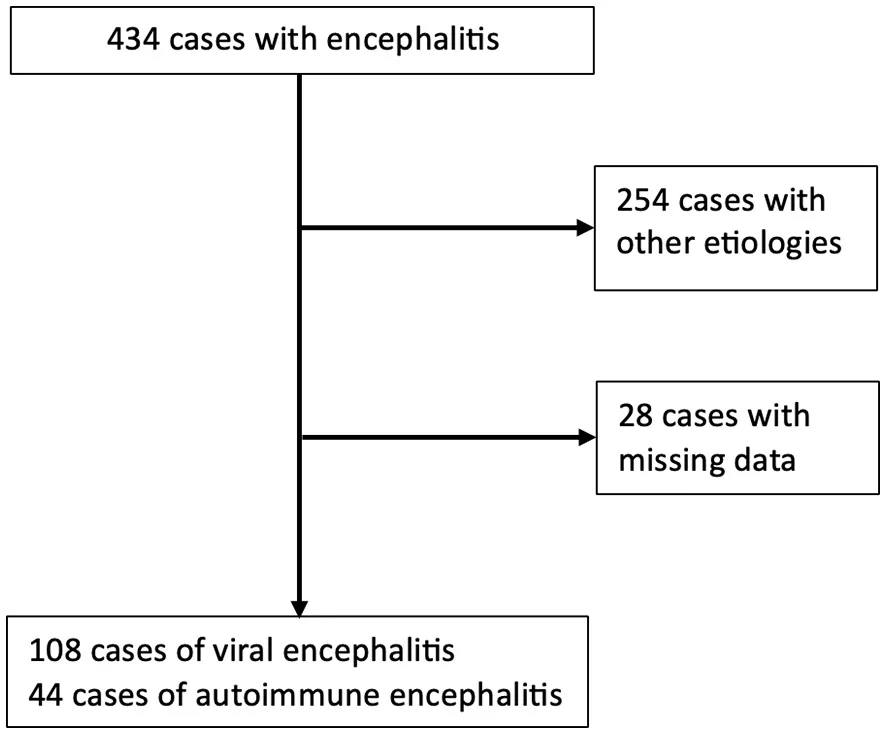
Process of defining the study population. Of 434 cases, 108 cases with viral encephalitis including Tick-borne encephalitis (TBE), *Herpes simplex* virus encephalitis (HSV), *Varizella Zoster* virus encephalitis (VZV) and 44 with autoimmune encephalitis were included.

A total of 108 cases with VE caused by HSV, VZV or TBE and 44 cases of autoimmune encephalitis were included. The mean age of the patients was 55 ± 16 years (minimum 15 years, maximum 94 years) for VE and 50 ± 19 years (minimum 19 years, maximum 79 years) for AE. In the cohort of AE, more patients were female (48%) compared to the cohort of VE (27%) (Table 1). Univariate logistic regression identified sex, headache, fever, the age-adjusted CSF/serum albumin quotient, CSF white blood cell count, lactate and protein concentration as significant differentiators of AE and VE (*p* < 0.05; Table 2).

**Table 1:** Distribution of baseline characteristics, clinical presentation, and CSF parameters.

Parameter (at onset of disease)	VE	AE
Age, mean (SD)	55 (16)	49 (19)
Sex female, *n* (%)	33 (27)	22 (49)
Headache, *n* (%)	81 (66)	8 (18)
Fever, *n* (%)	75 (61)	3 (7)
Focal deficits, *n* (%)	66 (54)	22 (49)
Altered neuro-psychiatric status, *n* (%)	99 (81)	38 (84)
Epileptic seizures, *n* (%)	23 (19)	14 (31)
CSF WBC count in cells/μL, mean (SD)	138 (182)	24 (47)
CSF lactate concentration in mmol/L, mean (SD)	2.42 (0.58)	2.02 (0.81)
CSF protein concentration in mg/L, mean (SD)	885 (374)	617 (704)
CSF/serum albumin quotient in relation to the age-specific ULN, mean (SD)	2.18 (1.10)	1.52 (1.93)
CSF-specific OCB, *n* (%)	29 (28)	16 (40)

For nominal variables, percentages for each subgroup are provided, whereas for continuous variables, means and standard deviations are reported. For CSF-specific OCBs, percentages were calculated based on the subset of cases for which CSF OCB data were available. AE, autoimmune encephalitis; CSF, cerebrospinal fluid; OCB, oligoclonal bands; SD, standard deviation; ULN, upper limit of normal; VE, viral encephalitis; WBC, white blood cell.

**Table 2:** Univariate logistic regression analyses to identify predictors of autoimmune vs. viral encephalitis.

Parameter (at onset of disease)	OR (95% CI)	*P* value
Age	0.984 (0.964–1.004)	0.123
Sex	2.487 (1.200–5.155)	0.014
Headache	0.121 (0.051–0.286)	< 0.001
Fever	0.041 (0.012–0.142)	< 0.001
Focal neurologic deficits	0.800 (0.396–1.615)	0.533
Altered neuropsychiatric status	1.276 (0.499–3.259)	0.611
Epileptic seizures	1.737 (0.777–3.882)	0.178
CSF WBC count	0.975 (0.963–0.987)	< 0.001
CSF lactate concentration	0.244 (0.109–0.545)	< 0.001
CSF protein concentration	0.998 (0.997–0.999)	0.004
Age-adjusted CSF/serum albumin quotient	0.995 (0.991–0.999)	0.009
CSF-specific oligoclonal bands	1,574 (0.719–3.448)	0.257

CSF, cerebrospinal fluid; CI, confidence interval; OR, odds ratio; WBC, white blood cell.

### 3.2 Selection of parameters for the score

To develop a clinical score for differentiating AE from VE, candidate variables were selected based on clinical significance and univariate logistic regression analysis. We chose fever, headache CSF protein concentration and CSF WBC, these are also reported to significantly differ between AE and VE in literature (21). Those parameters differed significantly in our univariate analysis as well. CSF lactate concentration appeared clinically significant, but wasn't tested in other studies comparing AE and VE (21, 22). We decided to include this parameter, which is supported by significant results in univariate analysis.

The inclusion of age and sex as potential parameters was discussed but ultimately rejected, as both AE and VE are highly heterogeneous conditions in regard to both parameters. Their clinical relevance and reliability across the full spectrum of AE and VE were considered limited. Furthermore, for age, selecting a clinically meaningful cutoff value was challenging (20). Age-adjusted CSF/serum albumin quotient was not included, as it yields similar information as CSF protein concentration and CSF-specific oligoclonal bands (OCB) were not included, as they usually take several days to be tested. Focal neurologic deficits, epileptic seizures and altered neuropsychiatric status are not specific for either AE or VE and were therefore excluded as data in different cohorts including ours is heterogeneous (21–23).

### 3.3 Model performance and validation

Continuous predictors were dichotomized using optimal cut-off values determined by ROC analysis according to the maximum Youden index. This revealed optimal cut-off values for CSF WBC count at 23 cells/μL, for CSF protein concentration at 478 mg/L and for CSF lactate concentration at 2.02 mmol/L, which also seems clinically plausible. For practical reasons we defined 25 cells/μL, 500 mg/L and 2.00 mmol/L respectively. A Firth logistic regression was conducted to examine the predictors of AE, with variables including fever, headache, leukocyte count, lactate concentration, and protein concentration. These binary variables were coded in such a way that one always coded for the association with AE to yield positive coefficients in the subsequent regression analysis (no fever = 1, no headache = 1, leukocyte count < 25 cells/μL = 1, lactate concentration < 2.00 mmol/L = 1, protein concentration < 500 mg/L = 1). The model was statistically significant, Likelihood Ratio Test χ^2^ = 99.83, df = 5, *p* < 0.001, *N* = 152, indicating that the five clinical predictors discriminated between patients with AE and VE. The absence of fever (β = 2.38, 95% CI: 1.07–4.01, *p* < 0.001) and protein concentration < 500 mg/L (β = 2.26, 95% CI: 1.14–3.53, *p* < 0.001) were the strongest predictors for AE (Table 3). The absence of headache (β = 1.65, 95% CI: 0.46–3.03, *p* = 0.006) and CSF leukocyte count < 25 cells/μL (β = 1.72, 95% CI: 0.65–2.87, *p* = 0.002) showed smaller significant associations with AE. CSF lactate concentration < 2.00 mmol/L emerged as the weakest and only non-significant predictor (β = 0.80, 95% CI: −035–1.95, *p* = 0.171). VIF values below 1.13 indicated that multicollinearity between the five parameters was not a confounding factor in the regression model. Internal validation using bootstrapping with 1,000 replicates demonstrated excellent discrimination with an optimism-corrected AUC of 0.93 (95% CI: 0.89–0.98), signifying that the model can discriminate between AE and VE patients with 93% accuracy. An optimism-corrected calibration slope of 0.83, 95% (CI: 0.19–1.36) and the calibration plot (Supplementary Figure S1) indicated that the model tends to overfit, making predictions too extreme. However, the mean absolute error of 0.025 shows that the average deviation between predicted probabilities and real observations is 2.5%. The model shows good accuracy of the predictions with an optimism-corrected Brier score of 0.09, 95% (CI: 0.06–0.12).

**Table 3:** Multivariate logistic regression analyses with five parameters to develop the score.

Parameter (at onset of disease)	ß coefficient	OR (95% CI)	*p*-value
Headache (no/yes)	1.65	5.22 (1.59–20.61)	0.006
Fever (no/yes)	2.38	10.79 (2.96–55.33)	< 0.001
CSF WBC count	1.72	5.59 (1.91–17.68)	0.002
CSF lactate concentration	0.80	2.22 (0.71–7.05)	0.171
CSF protein concentration	2.26	9.63 (3.12–34.03)	< 0.001

CSF, cerebrospinal fluid; CI, confidence interval; OR, odds ratio; WBC, white blood cell.

### 3.4 Clinical sum score

Scaling the regression coefficients of the five predictors by dividing each by the smallest (lactate concentration, β = 0.80) and rounding to the nearest integer point resulted in the weights for the clinical sum score (Table 4). The simplified yet more practical sum score (values between 0 and 11 points) maintained the high discrimination of the regression model with an AUC of 0.94 (95% CI: 0.90–0.99, Figure 3). The optimal cut-off to distinguish between AE and VE as determined by the Youden-index was 5.5, meaning that scores of 6 and higher indicate a higher probability of AE and scores of 5 and lower indicate a higher probability of VE. At this threshold the sum score achieved:

**Table 4:** Score for the differentiation of AE and VE.

Parameter	Value
Absence of fever	3 points
Absence of headache	2 points
CSF WBC count < 25 cells/μL:	2 points
CSF protein concentration < 500 mg/L	3 points
CSF lactate concentration < 2.00 mmol/L	1 point
	Sum: 11 points

Score values of 6 or higher indicate AE to be more likely than VE. CSF, cerebrospinal fluid; WBC, white blood cell.

**Figure 3:**
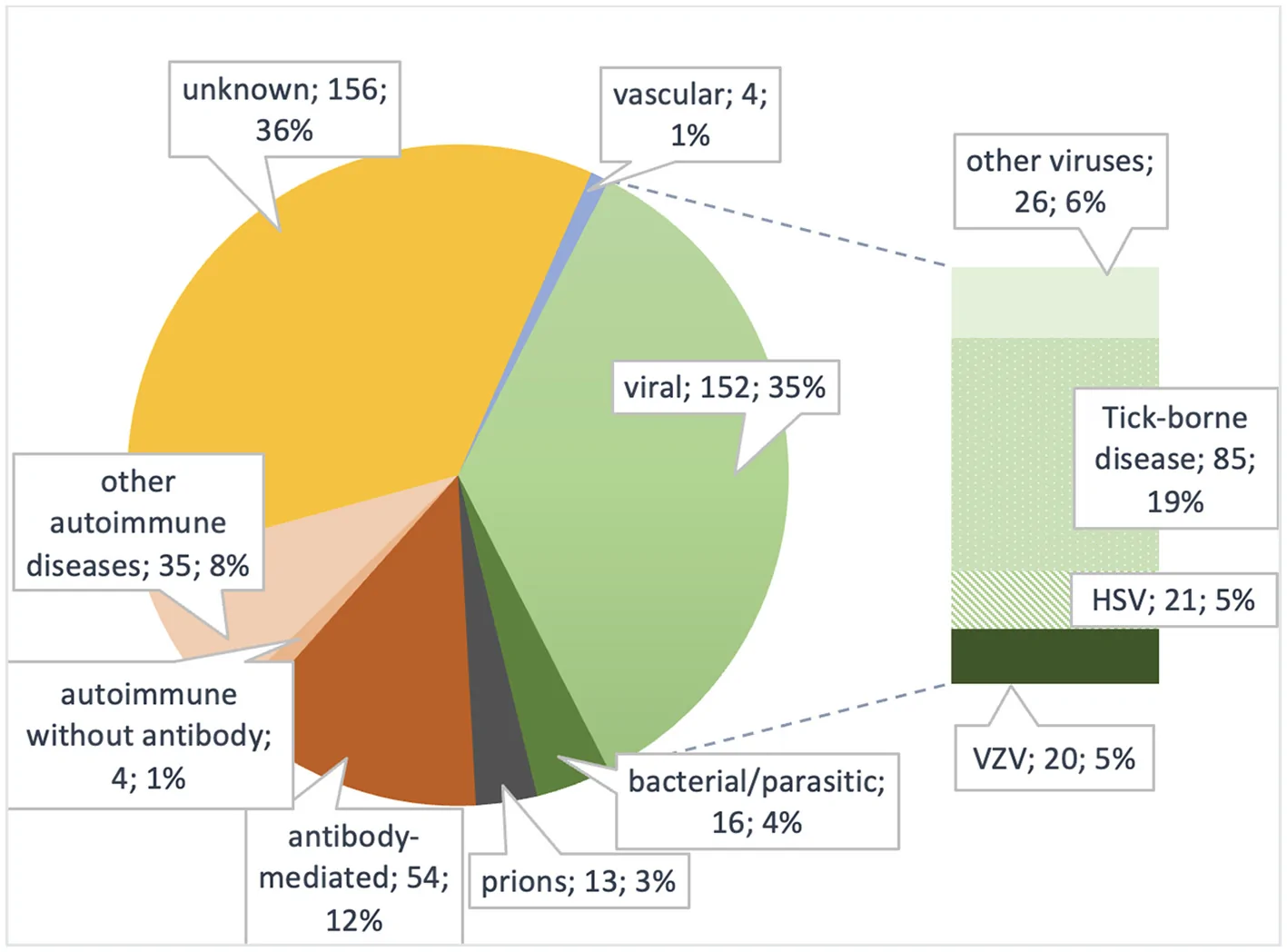
Receiver operating characteristic (ROC) curve of the clinical sum score. AUC, Area under the receiver operating characteristic curve.

- Sensitivity: 88.6% (95% CI: 77.3%−97.3%).
- Specificity: 87.0% (95% CI: 80.6%−92.6%).
- PPV: 73.6% (95% CI: 64.6%−83.3%).
- NPV: 95.0% (95% CI: 90.7%−98.9%).
- LR+: 6.84 (95% CI: 4.48–12.27%).
- LR-: 0.13 (95% CI: 0.03–0.25).

DCA was used to evaluate the clinical value of both the regression model and the sum score. The DCA curves (Supplementary Figure S2) show that the simplified sum score yields significant net benefit similar to the regression model.

### 3.5 Sensitivity analysis including age as metric variable

As defining a cut-off value for age was not possible based on our cohort and literature research and age did not improve overall score performance significantly, we did not include it in the final score. Nevertheless, we did a sensitivity analysis including the five initial parameters of the above-mentioned score and adding age as additional parameter. The overall model was highly significant (likelihood ratio test: χ^2^ = 106.50, df = 6, *p* < 0.001), and no relevant multicollinearity was observed (all variance inflation factors < 1.61). Internal validation demonstrated excellent discrimination (optimism-corrected AUC 0.94, 95% CI 0.91–0.98), good calibration (optimism-corrected calibration slope 0.80, 95% CI 0.12–1.30), and high overall predictive accuracy (optimism-corrected Brier score 0.08, 95% CI 0.05–0.11; mean absolute error 0.03). To maintain the linear relation between age and AE prediction whilst making make the score more practical in the clinic, age was categorized in steps of ten years. The beta coefficient for age was multiplied by ten accordingly. All regression coefficients were divided by the smallest coefficient (age, β = −0.51 per ten years) for scaling and rounded to the nearest integer point. Because age had a negative regression coefficient, meaning the probability for AE increases with younger age, we defined a cut-off age of >60 years based on the literature (20) as zero point. Younger age than the cut-off (in steps of ten years) was scored with positive points to ensure the final score maintained a positive range, which is easier to interpret in the clinic. This resulted in the following weights for the sum score (values between 0 and 19 points):

- Absence of fever: 4 points.
- Absence of headache: 4 points.
- CSF WBC count < 25 cells/μL: 5 points.
- CSF protein concentration < 500 mg/L: 4 points.
- CSF lactate concentration < 2.00 mmol/L: 1 point.
- Age per 10 years younger than 61: 1 point.

The optimal cut-off, determined by the Youden index, was 11.5 (score ≥12: AE more probable, score ≤ 11 VE more probable). At this threshold, the score demonstrated excellent diagnostic performance, with an AUC of 0.95 (95% CI 0.92–0.99), a sensitivity of 86.4% (95% CI 75.0–95.5%), a specificity of 92.6% (95% CI 87.0–97.2%), a positive predictive value of 83.0% (95% CI 73.1–92.5%), and a negative predictive value of 94.4% (95% CI 90.1–98.1%).

## 4 Discussion

Our study comprises a comparably large single center cohort of patients with definitive encephalitis. As reported in literature, VE is one of the most common entities in this group, with 35% of cases in our cohort slightly higher than 27% in the cohort of Granerod et al. (3), possibly due to the high frequency of TBE in Bavaria and our population. The proportion of autoimmune diseases (21%) aligns with that reported by Granerod et al., and the frequency of cases with no identified cause is comparable (36% vs. 37%). The latter group still is large and probably includes patients with undetected viral encephalitis (untested or even unknown pathogens) and cases of seronegative autoimmune encephalitis.

In our cohort, fever and headache were significantly more frequent in VE than in AE, consistent with literature, which reports headache in only 12% of patients with AE (24). Surprisingly, we did not find a significant difference in the occurrence of epileptic seizures between AE and VE, whereas these are reported to occur more frequently in patients with AE (25). This may be due to a high relative frequency of cases with HSV encephalitis in our cohort, which often is associated with epileptic seizures (26). Furthermore, our study focused on the first days of the treatment of patients, whereas epileptic seizures may develop in later stages of viral and autoimmune encephalitis. With respect to CSF findings, a wide range of results was observed in both VE and AE, likely reflecting the heterogeneity of these conditions. Taking only CSF WBC count into consideration, a distinction between AE and VE is not possible, as for example AE with N-methyl-D-aspartate (NMDA) receptor antibodies or glial fibrillary acidic protein (GFAP) antibodies may present with comparably high CSF WBC counts (3, 27, 28). Early treatment, however, is crucial both in cases of VE, especially HSV and VZV encephalitis, and autoimmune encephalitis (6, 29). As definite microbiological evaluation especially for ASI-based diagnosis is time-consuming, we developed a scoring system for early differentiation of AE and VE. Using two clinical and three CSF parameters, AE can be distinguished from VE with a sensitivity of 88.6% and a specificity of 87.0%. Brain MRI also plays a crucial role in the diagnostic evaluation of patients with encephalitis. However, as MRI is often not available within the first 24 h after presentation, it was not included in the present study or incorporated into the proposed score.

Our score does not address the possibility of other, less frequent causes of infectious encephalitis, such as bacterial, fungal or parasitic pathogens. Bacterial meningoencephalitis is often associated with CSF WBC count >1,000 cells/μL (30). In our subcohort of cases with VE, mean CSF WBC count was 140 cells/μL. In cases of very high CSF WBC count, bacterial (or fungal or parasitic) meningoencephalitis should be taken into consideration and treated accordingly.

Elevated CSF WBC count may have other causes, such as neoplastic meningitis or subarachnoid hemorrhage. These conditions typically present with distinct clinical features. We emphasize that our score is intended to be applied exclusively in cases of suspected viral or autoimmune encephalitis.

This is not the first attempt to develop a scoring tool for the differentiation of AE and VE, but other scores include exotic diagnostic tests (22) or more diagnostic parameters (23) with similar diagnostic power (in regard to AUC, sensitivity, specificity). Interestingly, in several models including ours, headache, fever and elevated CSF WBC count are associated with VE rather than AE (21–23). Regarding other parameters, for example focal neurological deficits (21) or CSF protein concentration (22) results are heterogeneous and, therefore, underline the importance of validation in larger cohorts.

The current study has limitations due to its retrospective design. Clinical symptoms were not evaluated systematically and therefore may be underreported in some patients. Furthermore, lumbar puncture was performed earlier in the clinical course of patients with VE than those with AE. In the latter cases, disease progression was slower and clinical signs sometimes less specific, therefore leading to delayed lumbar puncture. As this was a retrospective study, the diagnostic workup in individual cases did not follow a predefined diagnostic protocol, but was guided by the clinical judgment of the treating physicians of our tertiary referral center. In the subgroup of patients with encephalitis of unknown etiology, common causes of encephalitis (including HSV, VZV, TBE, and antibody-positive autoimmune encephalitis) were generally excluded. However, rare causes of encephalitis were not systematically investigated in all patients and may have been missed. Additionally, during the early phase of this study, some antineuronal antibodies had not yet been characterized. As a result, several cases were strongly suspected to be caused by AE, but lacked antibody confirmation. To improve the quality of the AE subgroup, these cases were excluded for the development of the score. Regarding the statistical analysis, several decisions were required to develop an appropriate statistical model and scoring system, including the selection of clinically relevant parameters for the score based on expert opinion and a review of the literature. However, this approach carries the risk that potentially informative variables, which may have demonstrated predictive value in other cohorts, were excluded from the model. In further studies we suggest to examine, if age as a parameter may improve the score. Furthermore, the logistic regression model yielded fairly wide 95% confidence intervals for the regression coefficients, indicating that the estimated effect sizes of the five predictors are imprecise and should therefore be interpreted with caution. Nevertheless, the model showed high discrimination and overall acceptable calibration, with an optimism-corrected AUC of 0.93 (95% CI 0.89–0.98), a Brier score of 0.09 (95% CI 0.06–0.12), and a calibration slope of 0.83 (95% CI 0.19–1.36), suggesting good overall predictive performance. The fact that the simplified sum score maintained excellent discrimination and favorable diagnostic characteristics supports the robustness and practical relevance of the underlying predictive model. Other major limitations of our study are the monocentric approach and the high frequency of TBE in our cohort which is endemic and the most frequent single cause of encephalitis in South Germany (2). The score, therefore, needs to be validated further in a multicenter and multiregional prospective study.

Our study highlights that VE and encephalitis of unknown etiology are the most frequent forms of encephalitis. Our scoring system incorporating headache and fever, CSF white blood cell count, CSF protein concentration, and CSF lactate concentration may help to distinguish patients with AE from those with VE. It is premature to draw therapeutic conclusions from the current data. However, following validation and further evaluation, the proposed score may become a valuable tool for clinical decision-making in routine practice.

## Data Availability

The raw data of this publication will be made available upon reasonable request.

## Supplementary material

Supplementary Figure S1 Calibration plot of the regression model.

Supplementary Figure S2 Decision curve analysis (DCA) of the regression model and the sum.

Supplementary Table 1 Detailed description of associated antibodies, autoimmune diseases and infectious pathogens in our cohort, given in absolute numbers.
