# Supplementary material for: Early differentiation of viral encephalitis and autoimmune encephalitis by clinical and basic laboratory data
Source: Front Neurol. 2026 Oct 13;17:1827372. doi: 10.3389/fneur.2026.1827372 (PMC13710236; doi:10.3389/fneur.2026.1827372)
Supplement: Supplementary file 1 [file Table_1.docx]

**Early differentiation of viral encephalitis and autoimmune encephalitis by clinical and basic laboratory data**

**Supplementary Table 1:** Detailed description of associated antibodies, autoimmune diseases and infectious pathogens in our cohort, given in absolute numbers.

| underlying pathogen or disease causing encephalitis | absolute number of cases |
| --- | --- |
| **Antibody mediated encephalitis (n = 54)** | |
| N-methyl-D-aspartate (NMDA) receptor antibodies | 20 |
| leucine-rich glioma-inactivated 1 (LGI1) antibodies | 6 |
| glutamic acid decarboxylase-65 (GAD65) antibodies | 4 |
| voltage-gated potassium channels (VGKC) antibodies | 3 |
| Hu antibodies | 3 |
| Ma2 antibodies | 3 |
| contactin-associated protein-like 2 (CASPR2) antibodies | 2 |
| immunoglobulin-like cell adhesion molecule 5 (IgLON5) antibodies | 2 |
| Yo antibodies | 2 |
| metabotropic glutamate receptor 5 (mGluR 5) antibodies | 2 |
| collapsin response mediator protein 5 (CRMP V) antibodies | 1 |
| gamma-aminobutyric acid type B (GABA-B) receptor antibodies | 1 |
| glial fibrillary acidic protein (GFAP) antibodies | 1 |
| glycine receptor antibodies | 1 |
| amphiphysin antibodies | 1 |
| inositol-1,4,5-trisphosphate receptor type 1 (ITPR1) antibodies | 1 |
| zink finger protein 4 (Zic4) antibodies and voltage-gated potassium channels (VGKC) antibodies | 1 |
| **Other autoimmune diseases (n = 34)** | |
| Rasmussen encephalitis | 8 |
| autoimmune after infection | 4 |
| Morbus Behçet | 2 |
| sarcoidosis | 2 |
| systemic lupus erythematodes | 1 |
| multiple sclerosis | 1 |
| Autoimmune encephalitis without proof of antibodies, without improvement under immunosuppressive therapy | 16 |
| **Bacterial/parasitic encephalitis (n = 16)** | |
| *Listeria monocytogenes* | 4 |
| *Borrelia burgdorferi* | 3 |
| *Mycobacterium tuberculosis* | 2 |
| *Toxoplasma gondii* | 2 |
| *Mycobacterium avium* | 1 |
| *Treponema pallidum* | 1 |
| *Capnocytophaga canimorsus* | 1 |
| *Klebsiella species* | 1 |
| septic-focal encephalitis due to endocarditis | 1 |
| **Viral encephalitis (n = 152)** | |
| Tick-borne disease | 85 |
| *Herpes simplex* virus | 21 |
| *Varicella zoster* virus | 20 |
| John Cunnigham virus (JC-virus) | 11 |
| Borna disease virus 1 | 5 |
| *Cytomegalovirus* | 2 |
| Ebstein Barr virus | 2 |
| Human immune deficiency virus | 2 |
| *Influenza* virus | 2 |
| Mumps virus | 1 |
| Adenovirus | 1 |
| **Encephalitis with underlying vascular disease (n = 4)** | |
| Susac’s syndrome | 4 |
